# Dual-Mode Gold Nanoparticle-Based Method for Early Detection of *Acanthamoeba*

**DOI:** 10.3390/ijms232314877

**Published:** 2022-11-28

**Authors:** Cristina Pastrana, J. Rafaela L. Guerreiro, Monisha Elumalai, Carlos Carpena-Torres, Almudena Crooke, Gonzalo Carracedo, Marta Prado, Fernando Huete-Toral

**Affiliations:** 1 Ocupharm Research Group, Department of Optometry and Vision, Faculty of Optics and Optometry, Complutense University of Madrid, C/Arcos de Jalón 118, 28037 Madrid, Spain; 2 Food Quality and Safety Group, International Iberian Nanotechnology Laboratory (INL), Av. Mestre José Veiga, 4715-330 Braga, Portugal; 3 BioMark@ISEP, School of Engineering of the Polytechnic Institute of Porto, Rua Dr. António Bernardino de Almeida 431, 4249-015 Porto, Portugal; 4 CEB/LABBELS, Center of Biological Engineering, Minho University, Campus de Gualtar, Rua da Universidade, 4710-057 Braga, Portugal; 5 Weldon School of Biomedical Engineering, Purdue University, West Lafayette, IN 47907, USA; 6 Department of Biochemistry and Molecular Biology, Faculty of Optics and Optometry, Complutense University of Madrid, C/Arcos de Jalón 118, 28037 Madrid, Spain

**Keywords:** *Acanthamoeba*, *Acanthamoeba* keratitis, gold nanoparticles, colorimetric assay, color coordinates, optical detection

## Abstract

*Acanthamoeba* keratitis is an aggressive and rapidly progressing ocular pathology whose main risk factor is the use of contact lenses. An early and differential diagnosis is considered the main factor to prevent the progression and improve the prognosis of the pathology. However, current diagnosis techniques require time, complex and costly materials making an early diagnosis challenging. Thus, there is a need for fast, accessible, and accurate methods for *Acanthamoeba* detection by practitioners for timely and suitable treatment and even for contact lens user as preventive diagnosis. Here, we developed a dual-mode colorimetric-based method for fast, visual, and accurate detection of *Acanthamoeba* using gold nanoparticles (AuNPs). For this strategy, AuNPs were functionalized with thiolated probes and the presence of target *Acanthamoeba* genomic sequences, produce a colorimetric change from red to purple. This approach allows the detection of 0.02 and 0.009 μM of the unamplified *Acanthamoeba* genome by the naked eye in less than 20 min and by color analysis using a smartphone. Additionally, real samples were successfully analyzed showing the potential of the technology considering the lack of point-of-care tools that are mostly needed.

## 1. Introduction

*Acanthamoeba* spp. are free-living protozoa that live in environments, such as soil, freshwater, and even seawater. Direct contact with these organisms rarely results in infections. However, the existence of lesions or injuries in the eyes, nostrils, or skin, might favor the entrance of these opportunistic parasites into the body, causing severe infections [1,2]. The most common infections are Amoebic granulomatous encephalitis, cutaneous Acanthamoebiasis, and *Acanthamoeba* keratitis (AK), all involving serious consequences.

*Acanthamoeba* keratitis is an ocular infection characterized by pain, photophobia, epithelial defect, and edema and if not suitable treated may lead to permanent visual impairment or blindness [3,4]. Risk factors associated with the development of keratitis include trauma, damage to the ocular surface, and the use of contact lenses (CL) [5,6]. Numerous studies have shown the association between the use of contact lenses and the development of AK, which is considered the major risk factor. In developed countries, it is estimated that 85% of AK cases occur in contact lens wearers [6,7]. The increasing incidence of AK is mainly related to exposure to water during contact-lens wear (bathing, swimming), poor hygiene practices, ineffective contact-lens solutions, ineffective disinfection of storage cases, as well as the use of tap water to clean them increases the risk of infection [8]. Seal et al. estimated an AK incidence of one per 30,000 contact lens wearers in England, Europe, Hong Kong, and other areas where CL fitting and hygiene habits are similar [9]. AK is an aggressive and rapidly progressing pathology; therefore, an early and differential diagnosis is considered the main factor to prevent the progression and improve the prognosis of the infection. Currently, up to 23 *Acanthamoeba* genotypes have been identified [10] with the genotype T4 responsible for approximately 90% of AK cases [11,12], and *Acanthamoeba castellanii* and *Acanthamoeba polyphaga* are the most common isolated species [3]. Traditionally, the diagnosis techniques rely on *Acanthamoeba* cultures (still considered the gold standard technique), in vivo microscopy, or the molecular biology method, polymerase chain reaction (PCR) [13,14,15]. In vitro cultures may have a sensitivity between 0–70% and require a sample by corneal scraping or biopsy, a process that takes around 10 days to obtain a result [13]. Regarding in vivo microscopy, it does not require a tissue biopsy and the diagnosis can be made instantly. This technique can reach a sensitivity and specificity of over 90% when performed by experts, however, only *Acanthamoeba* cysts are well recognized [16]. PCR technique has a high specificity of 94–100% and a sensitivity ranging from 66.7 to 100% being able to detect low concentrations of amoeba [13,15]. Overall, these methods require complex equipment and/or trained personnel, which can be costly and time-consuming, slowing down the accurate diagnosis and treatment, therefore, compromising the prognosis.

The time to diagnosis is crucial to stop the progression of the infection, and avoid permanent lesions, even though at the early stages most of the patients are misdiagnosed as the signs and symptoms of *Acanthamoeba* keratitis are difficult to differentiate from other types of keratitis [17]. Thus, new diagnostic tools for an early and accurate assessment of the infection are needed.

The use of nanoparticles in the field of nanomedicine for diagnosing and treating diseases has been increasing over the years. Gold nanoparticles (AuNPs) are one of the most studied mainly due to their characteristics: facile bioconjugation with a variety of biomolecules, biocompatibility, and specific surface plasmon resonance (SPR). They have been used in a wide range of applications including drug delivery, bioimaging, phototherapy, and radiotherapy [18]. In this sense, AuNPs have been employed to localize cellular biomarkers associated with different diseases such as cancer or Alzheimer, facilitating their diagnosis or acting as a therapeutic target [19]. Additionally, AuNPs have been shown antiangiogenic and anti-inflammatory properties [20].

In recent years, colorimetric gold nanoparticle-based biosensors have been emerging for the detection of different DNA sequences applied to a wide range of applications, showing great sensing potential. The first reports on the detection of DNA were based on the optical properties of gold nanoparticles (AuNPs), which induce a color change upon their cross-linking, offering a simple, fast, and cost-effective detection system [21]. Colorimetric detection of DNA sequences with AuNPs was also based on non-crosslinking aggregation strategies [22,23]. All of the mentioned strategies take advantage of the unique optical features of AuNPs for the detection of different pathogens such as hepatitis B [24], *Salmonella* spp. [25], human papillomavirus [26] or the SARS-CoV virus [27] and other targets of interest such as invasive Zebra mussel species [28].

Different in vitro studies demonstrated that the conjugation of different agents to AuNPs induced an antiamoebic effect [29,30]. However, this is the first study that used the optical properties of AuNPs to develop a new approach to *A. castellanii* detection. In this work, we proposed a fast, affordable, and easy-to-use AuNP colorimetric tool for the early detection and monitoring of *Acanthamoeba*, to be used by practitioners for a timely prescription of suitable treatment and by contact lens users for a preventive diagnosis.

## 2. Results and Discussion

### 2.1. Gold Nanoparticles Synthesis and Loading

**Figure 1:**
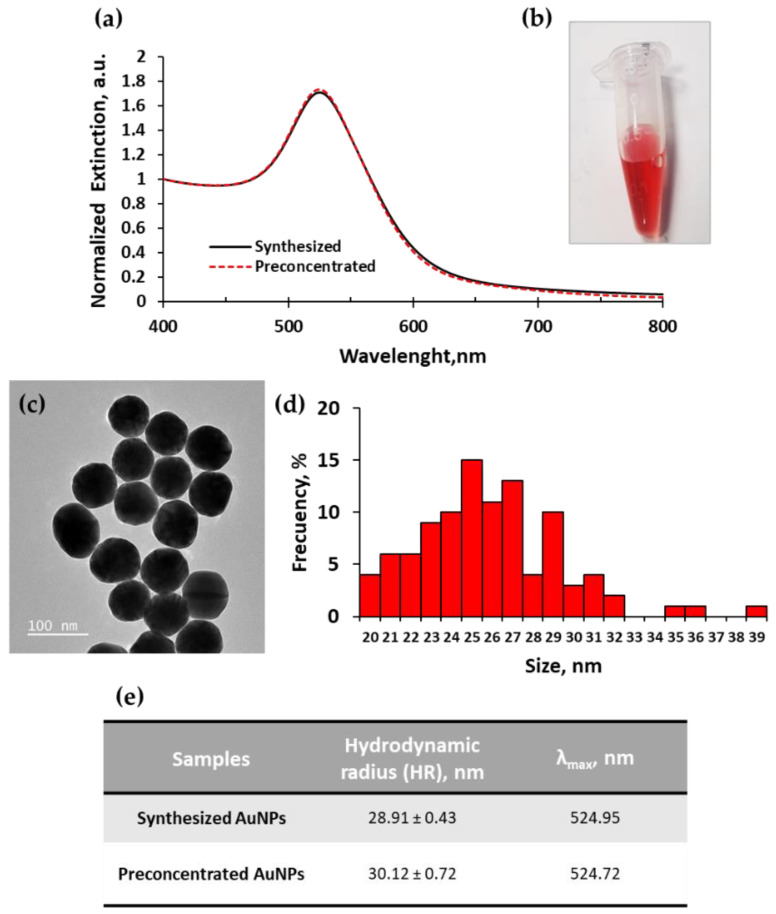
Characterization of AuNPs by different methods. UV-vis spectroscopy of synthesized and preconcentrated AuNPs (diluted 1000-fold) (**a**). Visual aspect of synthesized AuNPs, whose concentration was 0.43 nM (**b**). TEM image of preconcentrated AuNPs, scale 100 nm (**c**), and frequency of size calculated from TEM images (**d**). Hydrodynamic radius obtained from DLS and λ_max_ obtained from UV-vis of synthesized and preconcentrated AuNPs (**e**).

The detection strategy is based on the hybridization between the target DNA specific to *Acanthamoeba castellanii* and its complementary sequence loaded on two sets of AuNPs. The control of the synthesis conditions was essential to obtain high-quality monodispersed AuNP, assuring the stability and specificity of the colorimetric system. Thus, the synthesized AuNPs were characterized by UV-vis spectrophotometry, transmission electron microscopy (TEM), and dynamic light scattering (DLS). The UV-vis spectra for the synthesized and preconcentrated AuNPs display a single maximum extinction peak at 524.95 ± 0.04 nm and 524.72 ± 0.07 nm, respectively, indicating the presence of monodispersed nanoparticles of symmetric shape with a size around 20 nm, as shown in Figure 1a. The preconcentrated AuNPs were 524.72 ± 0.07 nm. The preconcentrated AuNPs showed no sign of aggregation upon centrifugation and can be considered stable given the tiny peak shift of 0.23 nm when compared to the synthesized AuNPs. Based on the spectra the estimated preconcentrated AuNPs concentration was 207 nM and the gold salt concentration [Au^0^] of 104 mM. The synthesized AuNP also presented a red color observed by the naked eye as displayed in Figure 1b.

TEM analysis of the synthesized AuNPs showed spherical-shaped particle morphology as shown in Figure 1c. Regarding their size, the frequency histogram of the transmission electron measurements showed a mean of 25.86 ± 3.50 nm, Figure 1d. DLS measurements showed a hydrodynamic radius of 28.91 ± 0.43 nm for synthesized AuNPs and 30.12 ± 0.72 nm for preconcentrated AuNPs (Figure 1e), a negligible difference between them of 1.21 nm that indicates the AuNPs remained stable. As expected, the size difference between TEM and DLS is mainly because the measurements by TEM were made in a dry state, while DLS measured the nanoparticles in solution meaning it accounts for the hydration layer around AuNP (due to the solvent molecules bound to the gold surface) [31].

Given the high quality of AuNPs obtained, we used the preconcentrated ones for DNA loading. Probe DNA loading onto the gold surface was carried out through two different approaches salt-aging and pH-assisted methods.

**Figure 2:**
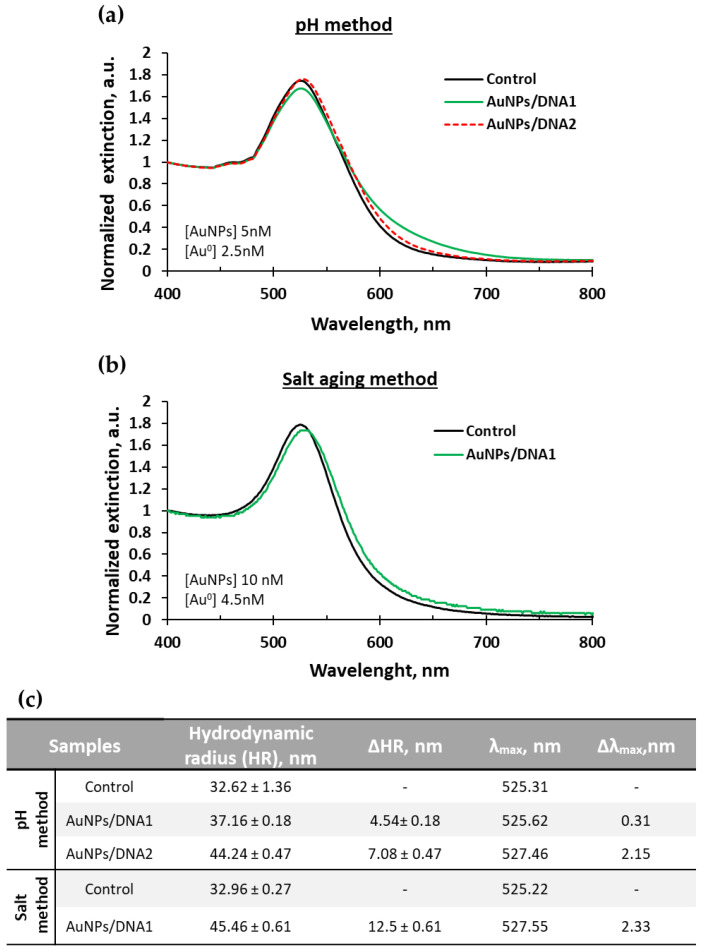
UV-vis spectroscopy of preconcentrated AuNPs alone (control) and loaded with DNA1 and DNA2 by pH method (**a**). UV-vis spectroscopy of preconcentrated AuNPs alone (control) and loaded with DNA1 by salt aging method (**b**). DLS data of concentrated AuNPs alone and loaded with DNA1 and DNA2 and the maximum peaks localization (λ_max_) obtained from UV-vis. The change in HR (ΔHR) and λ_max_ (Δλ_max)_ was made related to the control (**c**).

According to the literature, decreasing the pH allows DNA bases protonation favoring a faster loading of DNA onto AuNPs when compared to salt-aging and without requiring surfactants [32]. For this reason, initially, the low pH method was tested to load DNA1 and DNA2 on AuNPs. DNA2 loading on AuNPs resulted in a maximum peak absorbance at 527.5 nm, which means a shift of 2.15 nm compared to the preconcentrated AuNPs (control) as shown in Figure 2a. The shift in the position of the extinction peak represents the increase of the local refractive index around the nanoparticle due to the presence of DNA molecules on its surface. However, in the case of DNA1 a shift in the position of the extinction peak was not observed (non-significant peak shift variation of 0.31 nm) as seen in Figure 2a. This was further confirmed by DLS which showed a change of 4.5 ± 0.18 nm. Therefore, the results seem to indicate that this method is not suitable for the successful loading of DNA1 onto the AuNPs, which displayed a degree of aggregation. Regarding the data for DNA2 the extinction spectrum showed no aggregation, conserving the stability of AuNPs during the functionalization process, as confirmed by a similar spectrum of the control at the wavelength of approximately 650 nm. In addition, the presence of DNA on the surface was also confirmed by DLS, as shown in Figure 2c. The changes in hydrodynamic radius obtained after DNA binding showed an increase in diameter of 7.1 ± 0.7 nm for DNA2 compared to the control, which is within the expected increase of 5 to 10 nm for sequences with similar size.

Binding DNA to nanoparticles using a low pH-assisted method represents a simple and fast technique that only takes approximately 2 h. However, according to the results obtained for DNA1, the binding to nanoparticles was not as effective as for DNA2. This is highly related to the sequence composition and base protonation. Adenine and cytosine have pKa values higher than 3, which are protonated at pH 3, however, for guanine and thymine, the pH must be lower than 2 for protonation, which is outside of the experimental range [32,33,34]. The DNA1 has a slightly higher CT content (bases: A = 6, C = 5, G = 10, T = 7) when compared to DNA 2 (bases: A = 8, C = 6, G = 5, T = 9) making the loading harder with this method. Thus, DNA1 was loaded onto AuNP through the salt-aging method based on minimizing electrostatic repulsion. The salt-aging method is useful for most DNA sequences although it has the disadvantage of requiring longer incubation times (more than 12 h).

DNA1 loaded with the salt-aging method resulted in UV-vis spectra with no aggregation and a maximum peak extinction at 527.5 nm, which induced a shift of 2.33 nm and an increase of the hydrodynamic radius of 12.5 nm, Figure 2b,c. This method was more effective for DNA1 loading.

For this reason, the factors involved in this aggregation were adjusted: the salt concentration in the buffer solution, the use of surfactants (SDS) in the buffer composition, and adjust the DNA/NP ratio. The final salt concentration of 0.1 M, ratio DNA/AuNP 1150, and AuNPs concentration of 4 nM and 7 nM with an incubation time of 40 h showed aggregation of the samples. To avoid aggregation, a solution of SDS 10% + 100 mM PB was used. The final salt concentration of 0.18 M, ratio DNA/ AuNP of 4000, and AuNPs concentration of 4 nM and 10 nM with an incubation time of 12 h showed good results. The salt-aging method required more time and more excess DNA compared to the pH method. However, it should be considered most of the procedures requires an excess of DNA because it is estimated that only 30% of the DNA added binds to 13 nm nanoparticles and only 5% in the case of 50 nm nanoparticles [35].

### 2.2. Colorimetric System and Optimizations

The detection strategy is based on the hybridization between the target and complementary sequence (DNA1 and DNA2 probes) immobilized onto the AuNPs surface. In the absence of the target, the solution with the two sets of DNA-AuNPs remains well dispersed showing a red color. However, when the target is present, it interacts with the complementary DNA probes on the surface of AuNPs, inducing specific aggregation and a change in the solution color from red to purple. When nanoparticles come into close contact, an electromagnetic field coupling occurs inducing a localized surface plasmon resonance (LSPR) shift to longer wavelengths. This effect is highly dependent on the interparticle distance, which determines the detection sensitivity of the colorimetric system [36]. If the interparticle gap between AuNPs is smaller than 2.5 times the AuNP diameter, the surface plasmon resonance of the individual AuNP may be affected by the dipolar interaction of neighboring AuNP, leading to changes in plasmon resonance extinction spectra [37]. The smaller the gap, the larger the near-field coupling effect, and consequently the induced peak shift increases [38]. In this case, the probe sequence is composed of 44 base pairs, which corresponds to a gap between AuNPs of approximately 18 nm in length. Due to the importance of the interparticle distance of AuNPs in the colorimetric system, AuNPs diameters and DNA length need to be designed to assure the AuNP are larger than the interparticle distance, in this case, AuNPs size was ~25 nm. Although to achieve a specific and significant colorimetric response, salt concentration, AuNPs, and probe parameters needed to be optimized.

**Figure 3:**
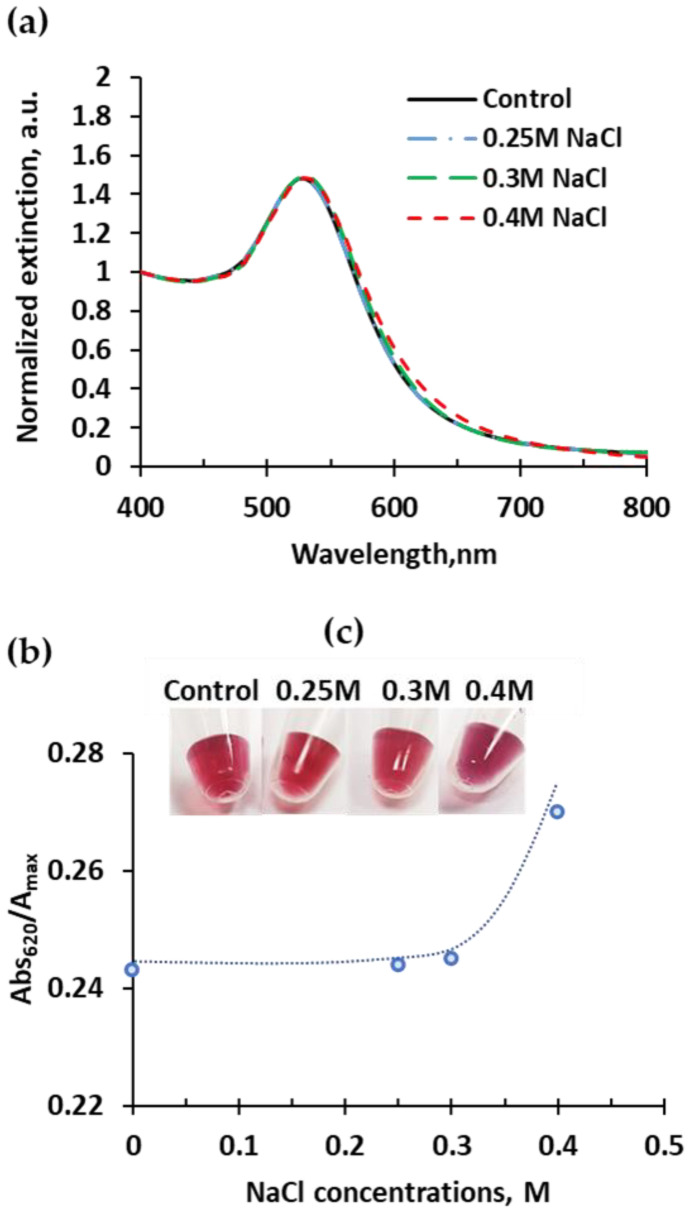
Effect of different salt concentrations (NaCl) on the stability of AuNPs loaded with the two DNA sequences (0.13 mM [Au^0^] and 2 nM [AuNPs]) by UV-vis spectroscopy (**a**). Estimated degree of aggregation (Abs_620_/Abs_max_) (**b**). Visual colorimetric change (**c**).

Salt concentration minimizes the electrostatic repulsion between the DNA strands bound to the AuNPs, in the solution and between DNA strands and the negatively charged surface, facilitating hybridization. High salt concentration increases the kinetics of the color response; however, it might also induce a non-specific aggregation of the AuNP compromising both the selectivity and sensitivity of the detection system. Therefore, salt concentration is an important factor that requires optimization. Salt concentration tests were carried out using a buffer composed of 2 M NaCl prepared in 10 mM, pH 7 PB buffer. The salt concentrations tested were 0.4, 0.3, and 0.25 M. The response obtained is shown in Figure 3. All samples were tested with a constant AuNP concentration of 2 nM. The samples with a salt concentration of 0.4 M showed a purple color, detectable by the naked eye, indicating the formation of AuNPs non-specific aggregates, Figure 3c. This aggregation was confirmed by UV-vis spectra with a slight increase on the right side of the LSPR maximum peak, Figure 3a. The degree of aggregation was estimated by the ratio Abs_620_ /Abs_max_, which corresponds to the two spectra regions influenced by dispersed or aggregated samples, Figure 3b. A salt concentration of 0.4 M resulted in the condition with the highest aggregation degree. Salt concentrations of 0.3 and 0.25 M NaCl preserve the colloidal stability of AuNPs, without aggregation, showing a red color and a stable degree of aggregation.

**Figure 4:**
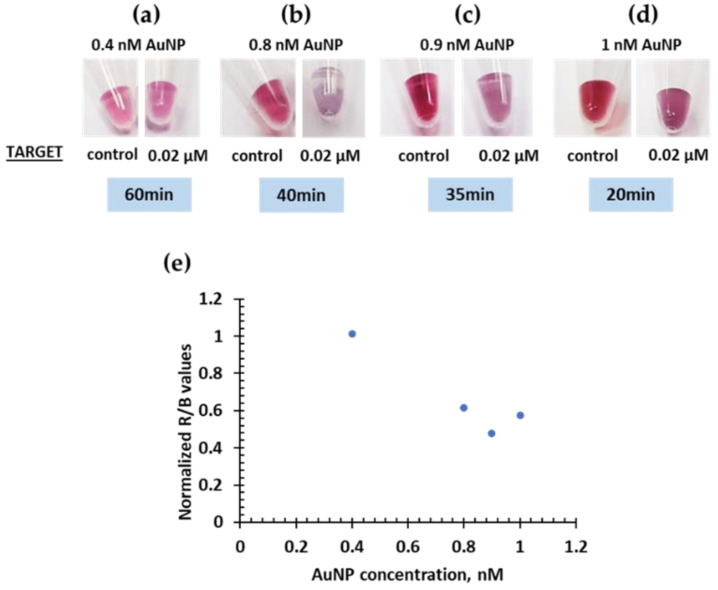
Effect of AuNP concentration on the colorimetric response: 0.4 nM (**a**), 0.8 nM (**b**), 0.9 nM (**c**), and 1 nM (**d**). Degree of aggregation based on ratio values (**e**).

The concentration of AuNP is also an important parameter to optimize as the color change might be easy to observe by the naked eye to a certain extent. The intensity of the red color is directly proportional to the concentration of AuNPs. Furthermore, the degree of AuNP aggregation in the colorimetric assays depends on the concentration of functionalized AuNPs. The concentration of DNA-AuNPs must be sufficient so that in presence of the target hybridizes and induces an optical response. Different AuNPs concentrations of 0.4, 0.8, 0.9, and 1 nM were tested (Figure 4). A visible change in the color intensity was noted for increasing concentrations. However, the time to achieve a visible color change increased for the lowest concentrations. For a concentration of 1 nM, the time to respond was about 20 min and increased from 30–40 min to 1 h with lower concentrations. For this reason, a concentration of 1 nM for DNA-AuNPs concentration was considered the best option for a rapid visible colorimetric response.

Different target concentrations were tested to assess the minimum concentration necessary to observe a color change. Theoretically, the minimum probe concentration to induce a significant color change is 4 DNA molecules per particle [39], in this case in this case, a larger ratio of target per AuNP was used to ensure aggregate formation. The hybridization tests were carried out with 1 nM for each set of DNA1 and DNA2-loaded AuNPs. The presence of the target in the samples triggers a color change from red to purple. Aggregation enhances the extinction at the blue wavelength (~620 nm); simultaneously the extinction peak decreases at the red wavelength (~520 nm) observed in UV-vis spectra.

**Figure 5:**
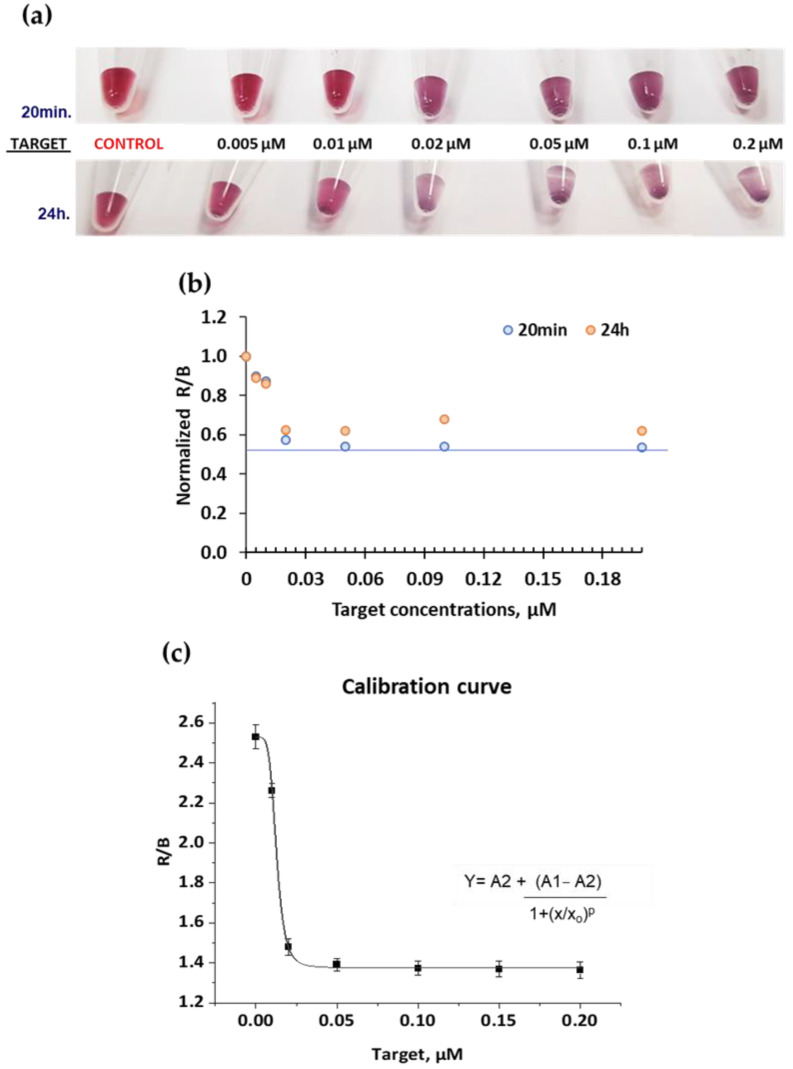
Colorimetric response obtained for different target concentrations ranging from 0.005 to 0.2 μM at a constant of 1 nM AuNP concentration for each DNA-AuNP set: Visible colorimetric change (**a**), R/B values (**b**), and calibration curve (**c**).

The visible color change after the addition of the different target concentrations is shown in Figure 5. After 20 min upon target addition, aggregation was visible by the naked eye for the concentrations of 0.02, 0.05, 0.1, and 0.2 μM and after 24 h the color change was even more significant. Lower target concentrations of 0.005 and 0.01 μM did not show a change of color compared to the control at 20 min, however, after 24 h, a slight color change seems to appear compared to the control. Based on these results it is observed that the minimum target concentration needed to obtain a visible short-term color change is 0.02 μM (1.12 × 10^11^ copies), which is considered the visual limit of detection (LoD) of the system. This value is equivalent to 0.2693 ng/μL.

An alternative for DNA target quantification is the RGB coordinates analysis of the digital images for accurate detection without the subjectivity inherent to the naked eye. To assess aggregation formation, the ratio of red and blue coordinate values (R/B) was calculated, and results were normalized based on the control (without target) for data comparison. concentration. Increasing target concentration starts to induce aggregation, which enhances the contribution of the component blue of the sample while it slightly reduces the red component, which was previously confirmed by UV-vis, therefore the ratio R/B decreased (Figure 5b). Along the time, as the aggregation increased, the same tendency was observed for both 24 h and 20 min.

The target DNA concentration (x) versus ratio R/B (y) assumes a non-linear behavior, as displayed in Figure 5c, fitted by the sigmoidal equation of Origin software shown in the graphic, where the curve parameters are A1 = 2.5293, A2 = 1.37643, x_0_ = 0.01268, and *p* = 5.03216. Three digital images (*n* = 3). were analyzed to check the stability of the system. The LoD using RGB coordinates was calculated based on three times the standard deviation of the control, which had a value of 0.009 μM (5.03 × 10^10^ copies). Compared with the LoD obtained by the naked eye (0.02 μM), the RGB approach offers a 2-fold increase in sensitivity and reduces the user subjectivity.

### 2.3. Real Samples Testing

**Figure 6:**
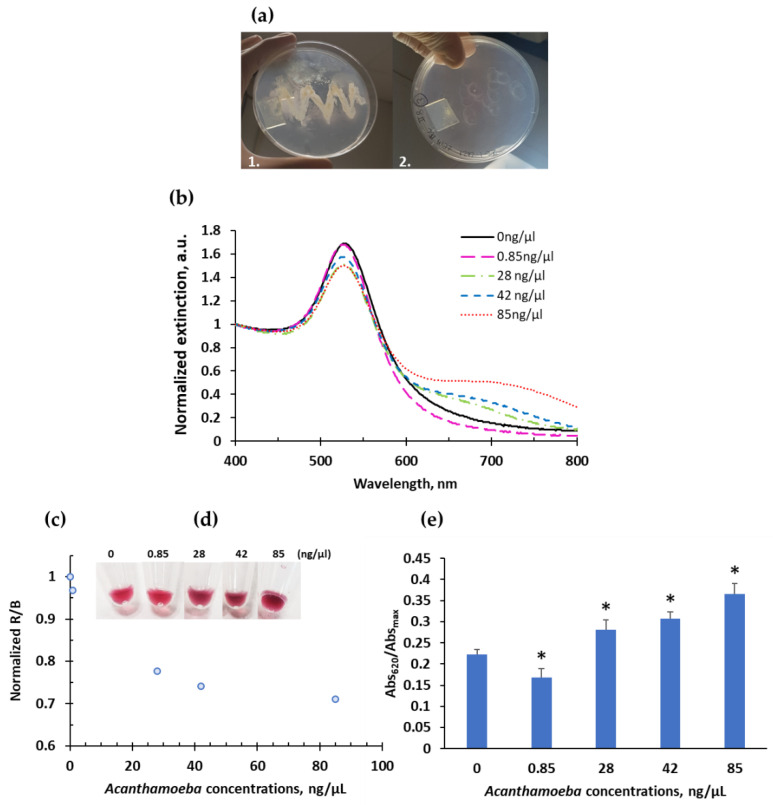
*A. castellanii* cultures fed with *E. coli* (**a1**) and after amoeba grew and ate all the *E. coli* (**a2**). Colorimetric response of *A. castellanii* real samples: UV-vis spectroscopy (**b**), R/B values (**c**), visible colorimetric response (**d**), and estimated aggregation degree (Abs_620_/Abs_max_) (**e**). * *p* < 0.05, Mann–Whitney U test, pairwise comparison with the control (0 ng/µL).

The optimized system tested with the synthetic *Acanthamoeba castellanii* probes was applied for the detection of *Acanthamoeba* in real samples. First, the *Acanthamoeba castellanii* was cultured in a Petri dish and feed with *E. coli.* As shown in Figure 6a, we can see the plates before and after the growth of amoebas. The final concentration of *Acanthamoeba* samples extracted from the cultures was adjusted to 5 × 10^5^ amoebas/mL. After performing the extraction protocol, total nucleic acids were quantified by nanodrop (*n* = 3), obtaining a ranging value from 400 to 500 ng/μL. From a sample of 485 ng/μL, dilutions were made to perform hybridizations. It is important to note that nanodrop provides a concentration value of total nucleic acids while this system is specific for a specific target sequence of *Acanthamoeba castellanii*. Thus, the concentrations of real samples can be used as a reference but cannot be directly compared with the concentrations reached with this system.

The results were analyzed based on the naked eye response, evaluation by UV-vis, and color coordinate analysis. The results by UV-vis showed a clear increase in aggregation at longer wavelengths for increasing concentrations of *A. castellanii*, which is visible for the 3 highest concentrations (28, 42, and 85 ng/μL), (Figure 6b,d,e). The degree of aggregation is also clear in Figure 6e, which tends to increase with increasing concentrations of amoeba. Similarly, the analysis of the R/B coordinates also tends to decrease with increasing concentrations of amoeba, as expected, showing a tendency to stabilize for higher concentrations indicating a possible signal saturation, Figure 6c. According to these results, the minimum concentration of *Acanthamoeba castellaniii* in a real sample to observe a visible change is less than 28 ng/μL, 295 amoebas/ μL. Obtained R/B ratios were used to determine unknown concentrations of real samples through the parameters of the calibrated sigmoidal curve fit (Table 1). Thus, a concentration of 28 ng/μL corresponds to 0.014 μM, which approximately coincides with the visible LoD of the system of 0.02 μM. This LoD, 0.2693 ng/μL (0.02 μM) is a hundred times lower than the minimum visible concentration of 28 ng/μL. It can be explained considering that nanodrop determines the total nucleic acids concentration and cannot distinguish sequences of interest from all others. Our system detects a much smaller amount of DNA molecules that would correspond to the specific DNA sequence targeted, while the nanodrop quantifies the total nucleic acids of the sample.

Compared to another simple system that avoids amplification methods [28,40,41,42,43] which adds complexity to the system, in primer design and applicability to the user, Table 2 shows other studies using colorimetric AuNPs-based systems for unamplified DNA detection of different pathogens. Similar LoD and response times to this work, have been reported in the detection of Sars-CoV [27] and *Mycobacterium* spp. [44]. On the other hand, while Bakthavathsalam et al. [45] and Yeung et al. [25] obtained higher LoD for *E. coli* and *Salmonella* spp. detection, other studies reported lower detection limits [46,47].

Currently, the main *Acanthamoeba* detection method based on nucleic acid detection is PCR. This technique has good sensitivity and has been only reported for DNA *Acanthamoeba* detection. Thompson et al. [15] reported a LoD of 11.3 DNA copies /10 μL being able to detect 0.7 cysts and 2.3 trophozoites per 10 μL of the reaction solution. However, the PCR technique must be performed by qualified personnel with bulky material in a laboratory, and getting a diagnosis can take from 1–2 to 5 days if it is not made in a special center or previous cultures are needed. For this reason, it is not the first option for diagnosis [13,48]. *Acanthamoeba* keratitis is an aggressive pathology, and when patients go to the clinic is because they already have symptoms, for this reason, is essential to make a fast and correct diagnosis to treat the pathology. Here, it is proposed a simple alternative without complex equipment for the readings so it can be used at the optometrist/ophthalmologist’s office for a timely diagnosis and suitable treatment. Our system is a great advantage as correctly there is no available tool for fast diagnosis and monitoring of *Acanthamoeba*. In this sense, the AuNPs system offers the possibility to detect the presence of *Acanthamoeba* by the users before symptoms appear, avoiding the infection or confirming the pathology.

## 3. Materials and Methods

### 3.1. Materials and Reagents

Gold (III) chloride trihydrate (HAuCl_4_·3H_2_O), sodium citrate tribasic dihydrate, sodium citrate dihydrate, sodium hydroxide (NaOH), disodium hydrogen phosphate, sodium chloride (NaCl), nitric acid (HNO_3_), hydrochloric acid (HCl), sodium dodecyl sulfate (SDS), sodium dihydrogen phosphate dihydrate (NaH_2_PO_4_·2H_2_O), sodium phosphate dibasic (Na_2_HPO_4_), and citric acid were purchased from Sigma-Aldrich (Sigma-Aldrich, St. Louis, MO, USA).

Phosphate buffer (PB), 0.1 M, pH 7 was prepared with NaH_2_PO_4_·2H_2_O and the Na_2_HPO_4_ in Milli-Q grade water (MQ) followed by pH adjustment. Citrate buffer (CB), 80 mM, pH 3 was prepared with sodium citrate tribasic dihydrate and citric acid in MQ and adjusted with HCl. The salting buffer was composed of NaCl (2 M), SDS (0.01%), and PB (10 mM). The salt buffer for hybridization was composed of NaCl (2 M) and PB (10 mM). Aqua regia was prepared in the proportion of 1:3 of HNO_3_ to HCl. Synthetic DNA target and oligonucleotide sequences thiol modified at 3′and 5′end were purchased from Stabvida (Caparica, Portugal). All DNA sequences were prepared in autoclaved, DNase, and RNase-free ultrapure water.

### 3.2. Selection of Specific Acanthamoeba-Sequences

*Acanthamoeba castellanii* species, belonging to the T4 genotype was chosen because is the most related to keratitis cases. *Acanthamoeba castellanii* hsp70 mRNA sequences were searched in the Nucleotide database (https://www.ncbi.nlm.nih.gov/nuccore, accessed on 3 January 2020) and performed an alignment of multiple nucleotide sequences with the CLUSTAL W2 allgorithm using default parameters [49], to identify highly conserved regions. The next step was the design of *Acanthamoeba castellanii* Hsp70-specific oligonucleotide probes with the Primer Express^®^ v2 Software (Thermo Fisher Scientific, Waltham, MA, USA). The specificity of these probes against *A. castellanii* cDNA was analyzed by PCR and sequenced (Genomics Unit of the Complutense University of Madrid, Madrid, Spain). Sequence analysis of the PCR product using the NCBI BLAST platform (National Institutes of Health, Bethesda, MD, USA) revealed the highest percent identity (99%) with *A. castellanii* str. Neff high molecular weight heat shock protein (ACA1_ 387860). Finally, the probes selected (the two DNA sequences) complementary to each end of the target sequence were modified with a thiol (-SH) at the 5′ and 3′ ends and were used for AuNPs loading (Table 3).

To verify the developed system is specific for *A.castellanii* detection, a sequence similarity study with other related keratitis species was performed using BLAST software. In the developed AuNPs based detection system, the target must bind to both probes (DNA1 and DNA2) consecutively on the same molecule to promote AuNPs aggregation and induce the color change. Therefore, the alignment study was performed on both probe sequences together. No significant similarity was found with other species commonly related to keratitis.

### 3.3. Gold Nanoparticles Synthesis

Spherical AuNPs were synthesized based on the sodium citrate gold reduction method described by Turkevich [50]. All the glassware material used in the synthesis was cleaned with aqua regia for 30 min and rinsed thoroughly with Milli-Q grade water (MQ). Briefly, 500 mL of a chloroauric acid (HAuCl_4_) solution 0.24 mM was brought to a vigorous boil for 5–10 min while stirring at 450 rpm, in a round-bottom flask. Then, 50 mL of a warm (~60 °C) solution of sodium citrate, 40 mM was rapidly added to the gold solution under continuous stirring for 15–20 min inducing an immediate color change from yellow to a colorless solution. To preconcentrate AuNPs, as-synthesized AuNPs were centrifugated for 15 min at 3000 rpm, and the supernatant was separated from the formed pellet. The preconcentrated AuNPs were then loaded with the DNA1 and DNA2 strands.

AuNPs were characterized by UV-vis spectrophotometry, (Lambda 950, PerkinElmer, Waltham, MA, USA) transmission electron microscopy (TEM) (JEM-2100, JEOL Ltd., Tokyo, Japan) and dynamic light scattering (DLS) (SZ-100z, Horiba, Kyoto, Japan). The DLS samples were prepared and diluted to a final metallic gold [Au^0^] concentration of 0.03 mM estimated based on UV-vis data assuming the correlation Abs_400 nm_ of 1.2 corresponds to 0.5 mM of [Au^0^] [51]. TEM images were analyzed by Image J software (National Institutes of Health, Bethesda, MD, USA) and the diameters were estimated based on the analysis of at least 200 particles. To analyze the UV-vis spectra of each AuNPs sample, the maximum extinction peak was determined using MATLAB R2018b software (MathWorks; Natick, MA, USA) and employing Lorentz model fitting.

### 3.4. AuNP Functionalized with DNA

AuNPs were loaded with two different DNA sequences by the pH-assisted method for DNA2 and the salt-aging method for DNA1.

#### 3.4.1. pH Reduction Method

Briefly, this method consists of a solution of 138 µL of citrate buffer, 10 mM, pH 3, and 10 µL of DNA2, 100 µM prepared with MQ water. The solution was sonicated for 3 min followed by the addition of 13 µL of 347 nM AuNPs and incubated in a thermomixer in a horizontal position at 450 rpm, at 22 °C for 2 h. The DNA2 to AuNPs concentration ratio used was 1000 and the excess of free DNA was removed by centrifugation at 8000 rpm (1523 RCF) for 10 min and the pellet was redispersed in 100 µL of 10 mM PB, pH 7. The DNA2-AuNPs solution was diluted to a final concentration of 5 nM AuNPs ([Au^0^] of 2.5 mM).

#### 3.4.2. Salt Aging Method

The salt method described by Mirkin and co-workers [52] was optimized to achieve the maximum DNA loading on AuNPs. A solution of 5 μL of concentrated AuNPs (217 nM) was mixed with 10 μL of PB (10 mM, pH 7), 1 μL of SDS (10%), 44 μL of DNA1 (100 μM), and 40 μL of MQ and incubated for 20 min at room temperature (22 °C). The DNA to AuNP concentration ratio used was 4055.

After this time, a buffer solution containing 2 M NaCl, SDS 0.01%, and 0.01 M PB pH 7 was added in 1 µL increments until reached a final concentration of 0.18 M NaCl. After each addition, the solution was sonicated for 10 s and incubated for 20 min in a mixer at 22 °C and 350 rpm, in a horizontal position and protected from the light. After the last addition of salt, the solution was incubated overnight (12 h) under the same conditions. Excess-free DNA1 was removed by centrifugation at 8000 rpm (1523 RCF) for 10 min and the pellet obtained was dispersed in 100 µL of 0.01% SDS. The final concentration of AuNPs was 10 nM ([Au^0^] of 4.5 mM).

### 3.5. Colorimetric Assay Procedure

The colorimetric strategy was based on the specific aggregation of AuNPs induced by target hybridization with the two sets of AuNPs modified with DNA1 and DNA2. The presence of the target, a DNA sequence specific to *Acanthamoeba castellanii* was confirmed by a colorimetric change from red to purple. The stabilization of AuNP loaded with DNA was assessed by studying their non-specific aggregation, for that increasing NaCl concentrations ranging from 0.25 to 0.4 M were tested with a constant AuNP concentration of 1 nM for each set of DNA-AuNP. The color intensity and naked eye perceptibility of color change were tested by keeping the target concentration at 0.02 μM and different AuNP concentrations from 1 to 0.4 nM.

For target detection, AuNP functionalized with DNA1 and DNA2 (2.3 µL for DNA1-AuNPs and 4 μL for DNA2-AuNPs) were mixed with the target (volume adjusted to each final concentration tested) and 2.5 μL of 2 M NaCl prepared in PB 10 mM, pH 7 solution to obtain a final NaCl concentration of 0.25 M, to minimize electrostatic repulsion between AuNP and DNA. Increasing target concentrations were tested from 0.005 to 0.2 μM. Controls were prepared in the same way, using the equivalent volume of 10 mM of PB, pH 7 instead of the target. Upon target addition, the solutions were kept at room temperature (22 ^o^C) until a visual color change.

#### Dual Colorimetric Strategy Based on the Naked Eye and Smartphone Digital Analysis

The colorimetric response was assessed directly by the naked eye by comparing the color of the target sample with the control sample. In addition, samples were photographed using a smartphone Samsung S8 (Suwon, South Corea) with dual 12-MP, 2960 × 1440-pixel resolution at 507 ppi, and photos were taken at a 15 cm of distance. The color of each photographed sample was evaluated based on the analysis of the red, green, and blue color coordinates (RGB coordinates). These coordinates were collected from 5 different spots for each sample within the same image captured by the smartphone and measured using the software Irfan view. At least three images were analyzed. The coordinate average data and R/B ratio were calculated, and the values were normalized based on the control with no aggregation. The correlation of DNA concentration versus R/B ratio was plotted and its fitting was performed using Origin 2021 software (OriginLab Corporation, Northampton, MA, USA).

### 3.6. Microbial Culture Samples

*Acanthamoeba castellanii* (CCAP 1501/1A) expansion cultures were made on non-nutrient AGAR (NNA) prepared in a PAS (Page’s amoeba saline) solution and autoclaved. *A. castellanii* cells were seeded with *Escherichia coli* bacteria (*E. coli*) (ATCC: PTA-1980) and the cultures were incubated at 30 °C for three days. After the incubation, cultures were observed in the optic microscope showing amoeba in the active trophozoite phase and the inactive cyst phase, and were counted using a Neubauer hemocytometer (Neubauer- improved, Paul Marienfeld GmbH & Co., Lauda-Königshofen, Germany). Amoeba was removed from the agar surface of the plates by flooding with Page’s amoebal saline. Samples extracted were preserved at −80 °C in a buffer containing guanidine thiocyanate (RLT buffer, RNeasy 96 Kit, Qiagen, SAS, France).

### 3.7. Real Sample Analysis with the Colorimetric System

Before mixing *Acanthamoeba* real samples with AuNPs, nucleic acids from *A. castellanii* samples were extracted and purified using the traditional phenol-chloroform procedure [53]. This avoids the non-specific aggregation of the AuNPs caused by RLT lysis buffer composition. Briefly, phenol-chloroform-isoamyl alcohol reagent is added to the *A. castellanii* samples (suspended in RLT buffer lysis) in a ratio of 25:24:1. Samples were conserved in ice for 10 min and centrifugated. The supernatant was kept and mixed with sodium acetate 3 M (1/10 total volume) and absolute ethanol (2 times final volume) and then, was kept as described before. After this, the pellet (nucleic acids part) was kept and dispersed in 150 μL PB 10 mM, pH 7.

*A. castellanii* genetic material extracted from the cultures was tested using a final salt concentration of 0.25 M and a DNA-AuNP concentration of 1.8 nM for each set, the same conditions as the previous tests with known concentrations of DNA from *A. castellanii*. Samples were analyzed both by the naked eye, and RGB coordinates, as previously described. The total DNA of real samples was also evaluated using a UV-vis spectrophotometer NanoDrop 2000c (Thermo Scientific™, Waltham, MA, USA).

## 4. Conclusions

Currently, ultrasensitive, and specific laboratory diagnostic methods for *Acanthamoeba* are available, however, portable, fast and easy-to-use diagnostic devices accessible are critical for monitoring the rapid process of *Acanthamoeba* infections, even before the first symptoms appear. In this study, the developed system detects specifically the presence of *Acanthamoeba castellanii* at 0.02 μM by the naked eye in less than 20 min without requiring further steps or sophisticated instrumentation. To our knowledge, we report for the first time a colorimetric sensing strategy for the detection of *Acanthamoeba* using modified gold nanoparticles paving the way to universal access to screening tools for infectious agents. The proposed method can be used by practitioners and/or in decentralized and low-resource settings, facilitating a precise treatment prescription, and stopping disease progression and complications associated. In the future, such an approach could be used for contact lens users for the prevention of *Acanthamoeba* spp. as an easy-to-use and low-cost detection tool.

## 5. Patents

Pastrana Robles, C.; Guerreiro, JR.; Huete Toral, F.; Prado Rodríguez M.; Pastrana Castro L.; Colligris, B.; Carracedo Rodríguez, J.G. P202130709—Sistema de detección de parásitos oculares. 2021. Spain.

## Figures and Tables

**Table 1:** Estimation of DNA concentrations of *A. castellanii* real samples by nanodrop and using the RGB system to calculate DNA concentration based on the calibration curve. The number of amoebas was calculated based on culture counting.

Real Samples	R/B Values	Nanodrop	Calibration Curve	Culture Quantification
		DNA Concentration, ng/μL	DNA Concentration, μM	Amoebas/μL
1	2.30	0.85	0.010	15
2	1.84	28.00	0.014	295
3	1.76	42.00	0.015	776
4	1.69	85.00	0.015	1960

**Table 2:** Colorimetric AuNPs based assays for DNA detection without amplification methods.

Microorganism	Approach	Sample Type	Measurement	Limit of Detection	Analysis Time	Reference
*Sars-CoV*	CL * aggregation	Vero cell culture	UV-Vis	0.18 ng/μL	10 min	[27]
*Mycobacterium* spp.	NCL * aggregation	Goat feces	Naked eye	2 ng/μL	15 min	[44]
*E. coli*	NCL aggregation	Unamplified genomic DNA	Naked eye	~54 ng	30 min	[45]
		Unamplified enzymatic digested genomic DNA		11.4 ng		
*Salmonella* spp.	Surface hybridization	Feces	Naked eye	5 × 10^8^ CFU	30 min	[25]
*Salmonella* *enterica*	CL aggregation	Genomic DNA	Naked eye	37 fM	15 min	[46]
*MERS-CoV*	NCL aggregation	Nucleic acids	UV-Vis	1 pmol/μL	10 min	[47]

* CL: cross-linking; NCL: non -cross-linking.

**Table 3:** DNA sequences selected for the colorimetric detection strategy.

Sequence (5′-3′)	
Target	GAGCCCACCGCCGCTGCCATCGCGTACGGTCTCGGCAAGAAGTC
DNA1	ATGGCAGCGGCGGTGGGCTCTTTAAAA-SH
DNA2	SH-AAAATTTTGACTTCTTGCCGAGACCGTA

## Data Availability

The data used to support the findings of this study are available from the corresponding author upon request.
